# Changes in Average Sodium Content of Prepacked Foods in Slovenia during 2011–2015

**DOI:** 10.3390/nu9090952

**Published:** 2017-08-29

**Authors:** Igor Pravst, Živa Lavriša, Anita Kušar, Krista Miklavec, Katja Žmitek

**Affiliations:** 1 Nutrition Institute, Tržaška cesta 40, SI-1000 Ljubljana, Slovenia; ziva.lavrisa@nutris.org (Ž.L.); anita.kusar@nutris.org (A.K.); krista.miklavec@nutris.org (K.M.); katja.zmitek@vist.si (K.Ž.); 2 VIST—Higher School of Applied Sciences, Gerbičeva cesta 51a, SI-1000 Ljubljana, Slovenia

**Keywords:** sodium, salt, processed foods, food composition, food labelling, food supply

## Abstract

A voluntary gradual reduction in the salt content of processed foods was proposed Slovenia in 2010. Our objective was to determine the sodium content of prepacked foods in 2015 and to compare these results with data from 2011. Labelled sodium content and 12-month sales data were collected for prepacked foods (*N* = 5759) from major food stores in Slovenia. The average and sales-weighted sodium content, as well as the share in total sodium sales (STSS) were calculated for different food category levels, particularly focusing on processed meat and derivatives (STSS: 13.1%; 904 mg Na/100 g), bread (9.1%; 546 mg), cheese (5.1%; 524 mg), and ready-to-eat meals (2.2%; 510 mg). Reduced sale-weighted sodium content was observed in cheese (57%), a neutral trend was observed in processed meat and derivatives (99%) and bread (100%), and an increase in sodium content was found in ready meals (112%). Similar trends were observed for average sodium levels, but the difference was significant only in the case of ready meals. No statistically significant changes were observed for the matched products, although about one-third of the matched products had been reformulated by lowering the sodium level by more than 3.8%. Additional efforts are needed to ensure salt reduction in processed foods in Slovenia. Such efforts should combine closer collaboration with the food industry, additional consumer education, and setting specific sodium content targets (limits) for key food categories.

## 1. Introduction

Excess dietary sodium intake is a well-recognised modifiable risk factor of high systolic blood pressure and a major cause of chronic non-communicable diseases [1,2]. Daily salt intake in most countries varies between 9–12 g [3], which is well above the World Health Organization (WHO) recommendations of a maximum of 5 g salt. It is estimated that globally reducing sodium intake to the recommended level would prevent about 2.5 million deaths annually, which has led to the WHO member states agreeing to cut the global population’s sodium intake by 30% by 2025 [4]. Salt-reduction strategy programmes are now established in many countries. These programmes commonly include industry engagement (reformulation), setting sodium content targets for foods, consumer education, front-of-pack labelling schemes, and interventions in public institutions [5]. However, the efficacy of these programmes is not always systematically assessed. Decreasing the sodium intake in the population is considered the best verification of the efficacy of salt-reduction programmes. Major improvements by way of salt intake lowering are reported by a few countries where a long-term multifaceted approach was accompanied with strong political support. For example, a voluntary salt-reduction programme in the UK has led to a reduction in dietary sodium intake by 15% since 2003/2004 [6]. The programme involved collaboration with the food industry and media in addition to scientific and health professionals. Finland was also able to considerably reduce its population’s sodium intake by applying similar voluntary codes of practice as part of the North Karelia project [7]. Several approaches were used, including greater education on a healthy diet and compulsory warning label use for high-salt products in many food categories, which resulted in the food industry reformulating numerous products in a bid to avoid such labels.

Although average sodium excretion in 24 h urine is recognised as the gold-standard marker for measuring salt intake in the population [8], this method cannot identify (changes in) food sources of sodium. Therefore, additional assessments of the efficacy of salt-reduction programmes also include monitoring sodium content in the food supply, especially in processed foods. This is very important in countries where salt-reduction programmes include the food industry voluntarily lowering salt content. Such studies have been more or less systematically performed in several countries, including in the USA [9,10], Australia [11,12], New Zealand [13], Canada [14] and the United Kingdom [15]. Studies mostly focus on monitoring the sodium content of processed prepacked foods and sometimes restaurant foods [16].

The sodium content of processed foods varies between different food categories. From a public health perspective, reformulation should be especially stimulated in food categories that present the largest contribution to sodium intake either due to their very high sodium content or their high consumption level by the population. Apart from plain salt (used as a condiment), the most important food sources of sodium include processed meat, bread and bakery products, cheese, as well as ready-to-eat prepared meals [15,17,18,19].

In Slovenia, the dietary salt intake is around 12 g daily [20], which is more than double the WHO recommendations. In 2010, a national Action Plan to Reduce Salt was accepted with the goal of cutting the population’s salt intake to the recommended 5 g daily before 2020 [21]. Reduced salt intake is also a key objective of the recent National Programme on Nutrition and Physical Activity 2015–2025 [22]. In practice, the Action Plan entailed different activities, including increasing public awareness of the recommended salt intake and the risks of excess intake, as well as supporting food operators in gradually reducing the salt content of processed foods. A stepwise approach was taken to reduce salt in critical food categories contributing the most to people’s salt intake. It was proposed that the industry should gradually cut processed foods’ salt content in critical food categories by 3.8–5.8% each year. The first sodium content monitoring of prepacked foods occurred in 2011 using a sales-weighted approach [19]. This study showed that a robust and cost-effective approach to assessing prepacked foods’ average sodium content could be achieved by employing a combination of 12-month food sales data, as provided by food retailers and covering most of the national market, and a comprehensive food composition database compiled using food labelling data. The study also revealed that in most investigated food categories, the market leaders had lower sodium levels than the average of that particular category [19].

The objective of this present study is to investigate sodium levels in prepacked foods in the Slovenian food supply in 2015 and to compare these results with the data available for 2011. We aim to particularly focus on processed meat and derivatives, plain bread, cheese, as well as ready-to-eat meals. The assessment relied on both the average sodium content of available prepacked foods and the sales-weighted average sodium content.

## 2. Materials and Methods 

### 2.1. Collection of Data for 2015

Cross-sectional data on the nutritional composition of prepacked foods in the Slovenian food supply were collected during January–February 2015 in five shops (two mega markets, two supermarkets, and a discount market) of three major grocery chains with the largest nationwide shop networks (Spar, Mercator, and Hofer). Sampling was done in Ljubljana, Slovenia. In agreement with the retailers, all prepacked products with a unique European/International Article Number (EAN) barcode were systematically photographed and recorded in an online Composition and Labelling Information System (CLAS) database [23]. The database is supported by a specially developed computer application, which enables digital recognition of EAN codes to accelerate the database’s formation and avoid duplicate entries. The information collected included a product’s name, list of ingredients, nutritional values, packaging volume, price, and EAN barcode. For the purposes of this article, only sodium contents are reported. The CLAS database was further complemented with country-wide, 12-month sales data obtained from retailers. This data covered most of the national market and ensured proper data handling. These sales data refer to the national market and present food product sales for the 12 months prior to the data collection (January–December 2014). This was arranged on the condition that the results would not reveal any particular retailer’s sales data. The sales data were given in universal form, including the EAN number, product description, number of products sold per year, and the quantity of food (kg or L) per packaging. The matching of foods was performed using EAN numbers. For the many products with the same EAN number that were available in different stores, the sales data from different retailers were combined to obtain the overall national yearly sales data for a product. According to the protocol developed within the Global Food Monitoring Initiative (GFMI) by Dunford et al. [24], we collected food data for the following categories: fruit and vegetable juices; soft drinks; cordials; coffee and tea; electrolyte drinks; waters; bread; biscuits; cakes; muffins and pastry; cereal bars; noodles; breakfast cereals; pasta; maize (corn); rice; couscous; unprocessed cereals; chocolate and sweets; jelly; chewing gum; pizza; soup; ready meals; pre-prepared salads and sandwiches; cheese; yoghurt products; milk; cream; desserts; ice cream and edible ices; butter and margarine; cooking oils; canned fish and seafood; chilled fish; frozen fish; baby foods; meal replacements; vegetables; fruit; jam and spreads; nuts and seeds; processed meat and derivatives; meat alternatives; crisps and snacks; sauces; mayonnaise/dressings; spreads; as well as honey and syrups. Alcoholic beverages and food supplements were not included. The samples included all foods available in the selected grocery stores at the time of sampling and for which sales data were available. In the next stage, food categories with less than 10 products with a nutritional declaration (sodium content) available were excluded from the analyses (maize; couscous; chewing gum; pre-prepared salads and sandwiches; chilled fish; meal replacements; as well as honey and syrups). The total food sample comprised 8323 products with available sales data, of which 5759 (69.2%) had their sodium content labelled and were therefore used for the calculations of the average sodium contents.

### 2.2. Use of Data for 2011

The comparison was performed with previously reported cross-sectional data for 2011 [19], which were collected in four shops (one mega market, two supermarkets, and a discount shop) of the same three major grocery chains as those collected in 2015. To enable easier comparison, all products in the 2011 database were re-categorised in line with the GFMI protocol [24]. Results are reported only for 22 (sub)categories for which the data collection included a complete food category as defined by GFMI: fruit and vegetable juices; soft drinks; electrolyte drinks; plain bread; biscuits; cakes, muffins and pastry; noodles; plain pasta; rice; pizza; ready meals; cheese; yoghurt products; milk; cream; butter and margarine; canned fish and seafood; processed meat and derivatives; pasta sauces; mayonnaise/dressings; meat spreads; vegetable spreads. Such dataset comprised 3745 products, of which 1374 (36.7%) were labelled with sodium content and used for the calculations of the average sodium contents.

### 2.3. Calculation of Average Sodium Content per (Sub)Category

The average sodium content of available prepacked foods (SCA; in mg of sodium per 100 g/mL) and average sodium content of sold prepacked foods (SCS; mg per 100 g/mL) were calculated for selected food (sub)categories for 2011 and 2015, according to the previously reported protocol [19]. SCA values present the average sodium content of all products within a specific (sub)category for which sodium/salt levels were labelled. SCS values present the average sodium content of all products sold within a specific category for which sodium levels were labelled. Ratios between SCA and SCS (SAR values) were also calculated.

### 2.4. Share in Total Sodium Sales (STSS)

Share in total sodium sales (STSS) was calculated using the data for 2015. Using the data related to the content of food per packaging, we calculated the amount (kg/L) of each product sold per year. In the next stage, the total sodium content of products sold within a specific food category (kg) was calculated using labelled sodium levels and 12-month sales data. STSS was calculated as the ratio between the total sodium in all sold foods in the (sub)category and the total sodium in all sold foods in the sample.

### 2.5. Data Processing and Statistical Analyses

The data were processed and evaluated using the computer programs Microsoft SQL Server Management Studio V13.0, Microsoft Analysis Services Client Tools 13.0, Microsoft Data Access Components (MDAC) 10.0, Microsoft Excel 2013 (Redmond, WA, USA), the program tool CLAS V1.0 (Composition and Labelling Information System; Nutrition Institute, Ljubljana, Slovenia), and the XLStat statistical software package V19.01 (Addinsoft, Barcelona, Spain). For SCA, the 95% confidence intervals (95% CI) were calculated. SCS was given as an exact value and, therefore, no confidence intervals are presented. Given that the samples consisted of all available foods in the food supply in the selected stores, a comparison between years was performed directly by calculating the SCA (2015/2011) and SCS (2015/2011) ratios. For statistical evaluation of differences in the average sodium content of available prepacked foods, a *t*-test or Mann-Whitney U test (for non-parametric variables) was used. For statistical comparison of the matched products in processed meat and derivatives, plain bread, cheese, and ready-to-eat meals between the years of 2011 and 2015, a paired *t*-test was used. *p* < 0.05 was considered to be statistically significant.

## 3. Results 

SCA and SCS was calculated using the CLAS database with representative data on the availability of prepacked foods on the Slovenian market and 12-month, country-wide sales data for each product included in the database (see Table 1 for data on selected food categories, and Supplementary Table S1 for complete data). The food categories with the largest contribution to overall sodium sales (STSS >3%) were processed meat and derivatives (13.1%); vegetables (9.4%; particularly in canned vegetables: 9.1%); waters (9.4%); bread (9.1%); milk (8.6%); biscuits (6.6%); crisps and snacks (6.3%); cheese (5.1%); breakfast cereals (3.8%); and sauces (3.0%). The highest sodium average content (SCA) was observed in sauces (1131 mg/100 g; 95% CI: 877–1386 mg); processed meat and derivatives (984 mg/100 g; 95% CI: 910–1058 mg); crisps and snacks (787 mg/100 g; 95% CI: 742–833 mg); canned fish and seafood (659 mg/100 g; 95% CI: 478–840 mg); mayonnaise and dressings (580 mg/100 g; 95% CI: 512–648 mg); bread (546 mg/100 g; 95% CI: 512–580 mg); pizza (539 mg/100 g; 95% CI: 493–586 mg); cheese (524 mg/100 g; 95% CI: 480–567 mg); and ready-to-eat meals (510 mg/100 g; 95% CI: 469–551 mg). On the other hand, inclusion of sales data gave the highest SCS values for processed meat and derivatives (904 mg/100 g); crisps and snacks (804 mg/100 g); sauces (720 mg/100 g); pizza (519 mg/100 g); and bread (505 mg/100 g). The biggest differences between SCA and SCS values were observed in the categories of breakfast cereals (SAR: 7%); fruit and vegetable juices (14%); noodles (27%); as well as waters (321%).

**Figure 1:**
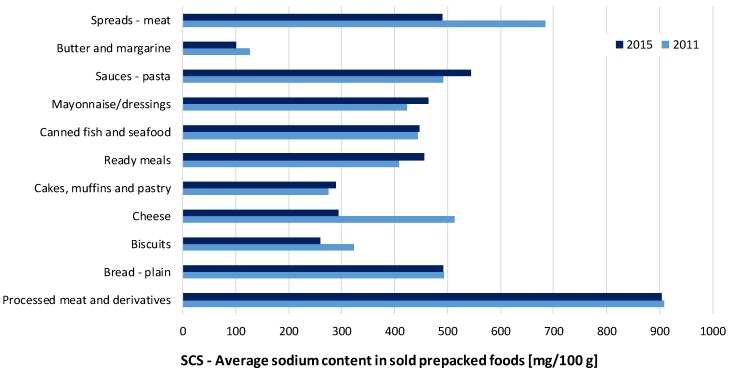
Comparison of the average sodium content of sold (SCS) prepacked foods for selected food (sub)categories (mg/100 g) in 2011 and 2015.

The comparison of the determined average sodium levels in prepacked foods in the Slovenian food supply in 2015 was further assessed according to the data collected in 2011 [19]. The between-years SCA/SCS levels are presented in Table 1, Supplementary Table S1 and Figure 1 (SCS levels only; included (sub)categories with STSS > 0.5%, for which data for both 2011 and 2015 were available). In the food (sub)categories making a notable contribution to overall sodium intake, reduced sale-weighted sodium content was observed in biscuits (SCS 2015/2011 ratio: 80%); cheese (57%); and meat spreads (71%), while there was a neutral trend in sodium content in processed meat and derivatives (99%) and bread (100%). Interestingly, increased salt content was also observed in a considerable number of food categories with comparatively lower STSS ratios, including cakes, muffins, and pastry (105%); ready-to-eat meals (112%); mayonnaise/dressings (109%); and pasta sauces (111%). Similar trends were also found for the SCA values of most of the abovementioned categories. However, statistically significant changes between the years of 2011 and 2015 were observed only for the ready-to-eat meals and pasta sauces. In both cases, we determined an increase in SCA levels (SCA 2015/2011 ratio: 106% (*p* = 0.013) and 155% (*p* = 0.002), respectively). We noted a considerable increase in the proportion of products with labelled sodium content from 2011 to 2015 (from 34% to 66% for ready-to-eat meals; as well as from 21% to 65% for pasta sauces). Due to a lack of sodium content data, a considerable proportion of 2011 sample was not included into analyses, and this limited the comparison of both samples in food categories with the lowest penetration of nutrition declaration (meat spreads (Percentage of products with labelled sodium content (% LSC): 4%); canned fish and seafood (12%); cakes, muffins, and pastry (12%); cheese (13%); processed meat and derivatives (15%); cream (19%); pasta sauces (21%); plain bread (30%); noodles (31%); and ready meals (34%)).

Considering the national Action Plan to Reduce Salt [21], which had proposed voluntarily gradually reducing the salt content of processed foods in critical food categories by 3.8–5.8% per year, progress in cutting salt content was particularly expected in processed meat and derivatives, plain bread, cheese, and ready-to-eat meals. Therefore, we further focused on these (sub)categories and compared the composition of matching products (same brands) for which composition data were available in both 2011 and 2015 databases. A total of 98 such foods were identified. No statistically significant changes were observed for the matched products in the selected food categories (*p* = 0.08, 0.22, 0.25, and 0.76 for processed meat and derivatives; cheese; plain bread; and ready-to-eat meals, respectively), although about one-third of the matched products had been reformulated by lowering the sodium level by more than 3.8% (35%, 32%, 24%, and 21%, respectively).

## 4. Discussion

Using a combination of 12-month food sales data provided by food retailers covering most of the national market along with a comprehensive food composition database compiled using food labelling data, we showed that processed meat and derivatives (STSS: 13.1%), canned vegetables (9.1%), and plain bread (8.1%) were major sources of salt in prepacked foods in the Slovenian food supply in 2015 due to their high sodium levels and high level of consumption by the population.

Similar to the 2011 study [25], the highest sodium content was found in processed meats and derivatives (SCS: 904 mg/100 g) in 2015. The between-years comparison shows that although the average sodium content in the category was reduced (SCA 2015/2011: 82%), this change was not significant (*p* = 0.13) and there were also no important changes in the sales-weighted sodium content (SCS 2015/2011 ratio: 99%). Reduced sale-weighted sodium content was observed in cheese, butter and margarine, biscuits, as well as meat spreads (SCS 2015/2011: 57%, 80%, 80%, and 71%, respectively) (Table 1 and Supplementary Table S1). Differences in SCA levels showed similar but insignificant trends (SCA 2015/2011: 84%, 86%, 95%, and 81%, respectively).

Bread is considered a major source of sodium intake in the Slovenian population [26]. This was confirmed by our study as bread contributed 9.1% of total sodium sales. Unfortunately, we did not observe any shift towards lower sodium levels, even though this food category was a particular target of activities within the national Action Plan to Reduce Salt [21]. There was no statistical significant change in the SCA of plain bread between 2011 and 2015 (109%; *p* = 0.12). Furthermore, the sales-weighted sodium content in plain bread was the same as that in 2011 (SCS: 492 mg/100 g). For comparison, about 10% lower sales-weighted sodium levels in bread was reported in the UK study [15]. Considering that bread in Slovenia is chiefly sold as a non-prepacked product (not included in this study), this limits the generalisability of our results for this specific food category. It should be mentioned that a step towards cutting sodium content in bread was suggested by a very recent initiative for the self-regulation of foods produced in the bakery product sector, which is expected to be launched by the Slovenian Chamber of Commerce (SCC) in 2018. Changing sodium levels in bread will be particularly challenging since a very large number of food businesses operate in this sector, many of which are not SCC members. An interesting approach was taken in the Netherlands, where the bakery sector itself requested that mandatory maximum sodium levels be set for bread to ensure fair competition, while the levels for other categories are voluntary and do not attract formal sanctions [27].

Increased sodium content was observed in cakes, muffins, and pastry; ready-to-eat meals; mayonnaise/dressings; and pasta sauces (SCS 2015/2011: 105%, 112%, 109%, and 111%), which altogether contributed about 7% of the total sodium sales (STSS). Similar trends were observed with the SCA ratios where there was a statistically significant increase in sodium content of ready-to-eat meals and pasta sauces (SCA 2015/2011: 106% and 155% with *p* < 0.05 for both). Given that such “convenience” foods are gaining in importance for a large number of consumers [28], greater efforts should be targeted at these food groups. Several European Union (EU) member states already use such an approach [3,6,29]; however, harmonised activities on the EU level are essential, particularly in those food categories featuring a large proportion of internationally produced foods. An example of such an activity would be setting international minimum targets for salt reductions in key food categories. This can be done within the “EU platform for action on diet, physical activity and health” [30] with the support of “the high level group on nutrition and physical activity”—a group of European government representatives led by the European Commission (EC) [31]. Surprisingly, bottled water was also identified as a major contributor to total sodium sales among prepacked foods (STSS: 9.4%). To gain greater insight into this, we identified high sodium mineral waters as a major contributor to this large share. Among the 80 waters included in our analyses, sodium content levels were available for 56 of them. Ten mineral waters high in sodium (sodium ≥ over 200 mg/L) represented 99% of the abovementioned STSS value. It should be noted that in most natural mineral sodium waters, the predominant ion accompanying sodium is bicarbonate, which is considered to have a smaller effect on blood pressure than equivalent amounts of sodium chloride [32,33]. Water with the highest sodium level in our database contained 1700 mg of sodium (and only 58 g of chloride) per litre. This particular mineral water was labelled with a warning that the recommended intake is up to 0.3 L per day.

In line with observations made in 2011 [19], sales-weighted sodium levels were lower than average sodium levels for most food categories in Slovenia, which suggests lower than average sodium contents in market leaders (Table 1). However, this could be attributed to a mix of different factors, including brand trust, price, taste and texture expectations, as well as consumer experiences with a particular food product, rather than consumers’ awareness of health risks created by a high sodium intake.

The first monitoring of sodium content in foods in the Slovenian food supply was performed just after the national Action Plan to Reduce Salt in 2011 was launched [21]. A limitation of the 2011 study [19] was that the food sample was originally collected to assess the penetration of different food labelling information, particularly nutrition and health claims [25]. Therefore, some food categories relevant to sodium intake were not included. However, a major strength of the 2011 data collection and of this present study is that all foods available in the selected food categories were included, while the varying importance (as dietary sources of sodium) of the different products was accounted for on a product-to-product basis by including sales data. Such an exact approach is rarely taken because researchers typically do not have access to sales data on a product-to-product basis. This approach was first taken in the UK by Mhurchu et al., who used a combination of commercial consumer panel food-purchasing data with nutrient data over 12 months for a more precise assessment of processed foods’ sodium content and estimation of the population’s exposure to sodium [15]. Since such a consumer panel does not exist in Slovenia, 12-month, nationwide sales data provided by retailers was used. The 2011 study protocol was revised for this present study to include all key prepacked food categories, resulting in a more extensive data collection. A strength of this present study is therefore the extent of the data collection and its employment of sales data to assess average sodium contents in various food categories. The database generated for such a study is useful for various purposes. Besides monitoring changes in the food supply, such a database can be also employed as a source of data in studies where food recalls or diaries are used. Considering that such a database should be regularly updated, this could present a considerable challenge in many countries. In addition, compiling such a comprehensive database is not always possible. Mutual trust must exist between academia and food retailers to successfully use our methodology (sales-weighted approach), which might not be feasible in all environments. In our case, such trust was gained with the national authorities’ support for our efforts, an open discussion of issues related to data sharing, and by our strict commitment to protect the data. Another limitation of the study is that about 30% of the selected products were excluded from further analyses due to missing data on sodium levels. The mandatory labelling of salt content on processed prepacked foods in the EU, which was enforced since December 2016 [34], will enable an even larger proportion of foods to be used in such studies in the future. Furthermore, the study was not designed to investigate non-processed foods, which are a notable source of sodium intake.

## 5. Conclusions

The results of this study indicate that activities forming the national Action Plan to Reduce Salt had a limited effect on sodium levels in major prepacked food categories that are considered as major dietary sources of sodium. In this respect, extra efforts are needed to ensure further progress, which should entail even closer collaboration with the food industry and additional consumer education. Specific target sodium content values should be set for key food categories, rather than the expected annual lowering of sodium content in foods, as contained in the existing Action Plan. Further studies are needed to verify the efficacy of salt-reduction programmes, which should include both measuring the dietary intake of sodium and changes in sodium levels in foods in the future food supply.

## Figures and Tables

**Table 1:** Average sodium content of available (SCA) and sold (SCS) prepacked foods for selected food categories in 2011 and 2015.

	Year 2015								Year 2011 ^6^			
Food Category	*N*	% LSC ^1^	Average Sodium Content (mg per 100 g/mL)		SAR: SCS/SCA Ratio ^4^	STSS ^5^	*N*	% LSC ^1^	Average Sodium Content (mg per 100 g/mL)		SCA Ratio 2015/2011	SCS Ratio 2015/2011
			SCA (95% CI) ^2^	SCS ^3^					SCA (95% CI) ^2^	SCS ^3^		
Waters	80	71%	15 (0–42)	47	321%	9.4%						
Bread	126	83%	546 (512–580)	505	92%	9.1%						
- plain	111	81%	530 (499–562)	492	93%	8.1%	155	30%	488 (449–527)	493	109%	100%
Biscuits	655	73%	335 (305–364)	259	78%	6.6%	485	45%	353 (312–394)	324	95%	80%
Cakes, muffins and pastry	285	71%	268 (246–289)	289	108%	2.5%	73	12%	226 (169–283)	275	119%	105%
Noodles	103	77%	103 (61–145)	27	27%	0.1%	67	31%	94 (0–195)	20	109%	136%
Breakfast cereals	212	94%	215 (182–248)	14	7%	3.8%						
Pasta	296	88%	128 (102–154)	75	58%	1.3%						
- plain	242	88%	61 (40–81)	55	91%	0.9%	281	53%	15 (7–23)	29	398% *	193%
- filled	54	87%	431 (380–482)	409	95%	0.4%						
Pizza	21	71%	539 (493–586)	519	96%	0.2%	23	70%	932 (649–1215)	799	58%	65%
Soups - concentrated	150	78%	425 (353–497)	387	91%	0.8%						
Ready meals	206	66%	510 (469–551)	457	90%	2.2%	152	34%	480 (367–579)	409	106% *	112%
Cheese	292	74%	524 (480–567)	294	56%	5.1%	381	13%	626 (429–823)	513	84%	57%
Butter and margarine	85	78%	144 (101–187)	101	71%	1.0%	74	62%	168 (100–236)	127	86%	80%
Canned fish and seafood	155	61%	659 (478–840)	447	68%	2.2%	180	12%	443 (375–511)	444	149%	101%
Vegetables	453	56%	395 (335–455)	319	81%	9.4%						
- canned	330	60%	484 (413–555)	382	79%	9.1%						
Processed meat and derivatives	362	47%	984 (910–1058)	904	92%	13.1%	363	15%	1116 (952–1258)	909	88%	99%
Meat alternatives	53	58%	453 (318–589)	220	48%	0.1%						
Crisps and snacks	206	86%	787 (742–833)	804	102%	6.3%						
Sauces	273	55%	1131 (877–1386)	720	64%	3.0%						
- pasta	108	65%	601 (528–673)	545	91%	1.1%	135	21%	386 (288–484)	492	155% *	111%
Mayonnaise/dressings	48	92%	580 (512–648)	464	80%	1.3%	36	47%	576 (481–671)	424	101%	109%
Spreads	234	57%	425 (359–490)	192	45%	1.0%						
- meat	131	45%	509 (469–548)	490	96%	0.6%	123	4%	626 (573–679)	686	81%	71%
- vegetable	48	71%	653 (454–853)	524	80%	0.1%	39	44%	578 (408–748)	459	113%	114%

Notes: Results for all food categories are presented in a Supplementary Table S1; ^1^ % LSC: Percentage of products with labelled sodium content; ^2^ SCA: Average sodium content of available prepacked foods (95% confidence interval); ^3^ SCS: Average sodium content of sold prepacked foods; ^4^ SAR: Ratio between SCA and SCS; ^5^ STSS: Share in total sodium sales; ^6^ Data from Korošec et al. 2014 [19], with foods categorised according to Dunford et al. 2012 [24]. * *p*-value < 0.05 (using *t*-test and Mann-Whitney test for comparison of Average sodium content of available prepacked foods (SCA) between both years).

## Supplementary Materials

The following are available online at www.mdpi.com/2072-6643/9/9/952/s1. Table S1: Average sodium content of available (SCA) and sold (SCS) prepacked foods for selected food categories in 2011 and 2015.
