# Supplementary material for: Changes in Average Sodium Content of Prepacked Foods in Slovenia during 2011–2015
Source: Nutrients. 2017 Aug 29;9(9):952. doi: 10.3390/nu9090952 (PMC5622712; doi:10.3390/nu9090952)
Supplement: Supplementary file 1 [file nutrients-09-00952-s001.docx]

**Supplementary Table.** Average sodium content of available (SCA) and sold (SCS) prepacked foods for selected food categories in 2011 and 2015.

|  | **Year 2015** | | | | | |  |  | **Year 2011** ^5^ | |  |  |  |
| --- | --- | --- | --- | --- | --- | --- | --- | --- | --- | --- | --- | --- | --- |
| **Food category** | ***N*** | **% LSC ^1^** | Average Sodium Content (mg per 100 g/mL) | | **SAR: SCS/SCA ratio ^4^** | **STSS: Share in total  Sodium sales** | ***N*** | **% LSC ^1^** | Average Sodium Content  (mg per 100 g/mL) | | **SCA ratio** ^6^ **2015/2011** | **p-value (SCA ratio)** | **SCS ratio 2015/2011** |
|  |  |  | **SCA (95% CI) ^2^** | **SCS^3^** |  |  |  |  | **SCA (95% CI) ^2^** | **SCS ^3^** |  |  |  |
| Fruit and vegetable juices | 243 | 74% | 21 (10–31) | 3 | 14% | 0,1% | 240 | 61% | 13 (7–19) | 2 | 160% | 0.774 | 122% |
| Soft drinks | 338 | 86% | 13 (11–16) | 14 | 109% | 2,8% | 376 | 49% | 20 (16–24) | 20 | 66% * | <0.001 | 71% |
| Cordials | 88 | 66% | 19 (11–27) | 11 | 58% | 0,1% |  |  |  |  |  |  |  |
| Coffee and tea | 456 | 23% | 175 (137–214) | 250 | 143% | 0,4% |  |  |  |  |  |  |  |
| - Coffee | 209 | 33% | 244 (199–290) | 265 | 109% | 0,4% |  |  |  |  |  |  |  |
| - Tea | 247 | 14% | 40 (0–87) | 51 | 127% | 0,0% |  |  |  |  |  |  |  |
| Electrolyte drinks | 13 | 100% | 104 (0–228) | 41 | 39% | 0,1% | 18 | 100% | 42 (31–53) | 24 | 247% | 0.869 | 172% |
| Waters | 80 | 71% | 15 (0–42) | 47 | 321% | 9,4% |  |  |  |  |  |  |  |
| Bread | 126 | 83% | 546 (512–580) | 505 | 92% | 9,1% |  |  |  |  |  |  |  |
| - plain | 111 | 81% | 530 (499–562) | 492 | 93% | 8,1% | 155 | 30% | 488 (449–527) | 493 | 109% | 0.116 | 100% |
| - other | 15 | 93% | 648 (506–790) | 630 | 97% | 1,0% |  |  |  |  |  |  |  |
| Biscuits | 655 | 73% | 335 (305–364) | 259 | 78% | 6,6% | 485 | 45% | 353 (312–394) | 324 | 95% | 0.493 | 80% |
| Cakes, muffins and pastry | 285 | 71% | 268 (246–289) | 289 | 108% | 2,5% | 73 | 12% | 226 (169–283) | 275 | 119% | 0.415 | 105% |
| Cereal bars | 56 | 96% | 135 (110–160) | 134 | 99% | 0,1% |  |  |  |  |  |  |  |
| Noodles | 103 | 77% | 103 (61–145) | 27 | 27% | 0,1% | 67 | 31% | 94 (0–195) | 20 | 109% | 0.858 | 136% |
| Breakfast cereals | 212 | 94% | 215 (182–248) | 14 | 7% | 3,8% |  |  |  |  |  |  |  |
| Pasta | 296 | 88% | 128 (102–154) | 75 | 58% | 1,3% |  |  |  |  |  |  |  |
| - plain | 242 | 88% | 61 (40–81) | 55 | 91% | 0,9% | 281 | 53% | 15 (7–23) | 29 | 398% * | <0.001 | 193% |
| - filled | 54 | 87% | 431 (380–482) | 409 | 95% | 0,4% |  |  |  |  |  |  |  |
| Rice | 95 | 78% | 18 (9–26) | 8 | 46% | 0,1% | 86 | 47% | 27 (15–39) | 20 | 67% | 0.248 | 41% |
| Unprocessed cereals | 138 | 66% | 49 (19–80) | 84 | 171% | 0,6% |  |  |  |  |  |  |  |
| Chocolate and sweets | 803 | 75% | 178 (0–388) | 130 | 73% | 2,7% |  |  |  |  |  |  |  |
| Pizza | 21 | 71% | 539 (493–586) | 519 | 96% | 0,2% | 23 | 70% | 932 (649–1215) | 799 | 58% | 0.060 | 65% |
| Soups - concentrated | 150 | 78% | 425 (353–497) | 387 | 91% | 0,8% |  |  |  |  |  |  |  |
| Ready meals | 206 | 66% | 510 (469–551) | 457 | 90% | 2,2% | 152 | 34% | 480 (367–579) | 409 | 106% * | 0.013 | 112% |
| Cheese | 292 | 74% | 524 (480–567) | 294 | 56% | 5,1% | 381 | 13% | 626 (429–823) | 513 | 84% | 0.971 | 57% |
| Yoghurt products | 362 | 94% | 48 (42–53) | 41 | 87% | 2,9% | 328 | 53% | 46 (40–52) | 42 | 104% * | 0.041 | 98% |
| Milk | 156 | 93% | 41 (38–44) | 46 | 113% | 8,6% | 83 | 59% | 40 (34–46) | 40 | 102% | 0.805 | 114% |
| Cream | 95 | 75% | 71 (51–92) | 47 | 66% | 1,2% | 47 | 19% | 58 (37–79) | 46 | 123% | 0.792 | 102% |
| Desserts | 148 | 67% | 71 (59–82) | 55 | 78% | 0,5% |  |  |  |  |  |  |  |
| Ice cream and edible ices | 179 | 80% | 55 (50–59) | 61 | 112% | 0,7% |  |  |  |  |  |  |  |
| Butter and margarine | 85 | 78% | 144 (101–187) | 101 | 71% | 1,0% | 74 | 62% | 168 (100–236) | 127 | 86% | 0.602 | 80% |
| Cooking oils | 190 | 67% | 3 (0–6) | 2 | 59% | 0,0% |  |  |  |  |  |  |  |
| Canned fish and seafood | 155 | 61% | 659 (478–840) | 447 | 68% | 2,2% | 180 | 12% | 443 (375–511) | 444 | 149% | 0.259 | 101% |
| Frozen fish | 50 | 56% | 333 (137–529) | 261 | 78% | 0,2% |  |  |  |  |  |  |  |
| Baby foods | 104 | 99% | 46 (26–65) | 23 | 51% | 0,0% |  |  |  |  |  |  |  |
| Vegetables | 453 | 56% | 395 (335–455) | 319 | 81% | 9,4% |  |  |  |  |  |  |  |
| - other | 123 | 45% | 72 (42–101) | 49 | 69% | 0,3% |  |  |  |  |  |  |  |
| - canned | 330 | 60% | 484 (413–555) | 382 | 79% | 9,1% |  |  |  |  |  |  |  |
| Fruit | 208 | 58% | 36 (20–51) | 23 | 65% | 0,1% |  |  |  |  |  |  |  |
| Jam and spreads | 167 | 41% | 16 (11–22) | 11 | 66% | 0,0% |  |  |  |  |  |  |  |
| Nuts and seeds | 139 | 49% | 287 (30–543) | 136 | 48% | 0,4% |  |  |  |  |  |  |  |
| Processed meat and deriv. | 362 | 47% | 984 (910–1058) | 904 | 92% | 13,1% | 363 | 15% | 1116 (952–1258) | 909 | 88% | 0.131 | 99% |
| Meat alternatives | 53 | 58% | 453 (318–589) | 220 | 48% | 0,1% |  |  |  |  |  |  |  |
| Crisps and snacks | 206 | 86% | 787 (742–833) | 804 | 102% | 6,3% |  |  |  |  |  |  |  |
| Sauces | 273 | 55% | 1131 (877–1386) | 720 | 64% | 3,0% |  |  |  |  |  |  |  |
| - other | 165 | 48% | 1602 (1150–2054) | 871 | 54% | 2,0% |  |  |  |  |  |  |  |
| - pasta | 108 | 65% | 601 (528–673) | 545 | 91% | 1,1% | 135 | 21% | 386 (288–484) | 492 | 155% * | 0.002 | 111% |
| Mayonnaise/dressings | 48 | 92% | 580 (512–648) | 464 | 80% | 1,3% | 36 | 47% | 576 (481–671) | 424 | 101% | 0.953 | 109% |
| Spreads | 234 | 57% | 425 (359–490) | 192 | 45% | 1,0% |  |  |  |  |  |  |  |
| - meat | 131 | 45% | 509 (469–548) | 490 | 96% | 0,6% | 123 | 4% | 626 (573–679) | 686 | 81% | 0.101 | 71% |
| - vegetable | 48 | 71% | 653 (454–853) | 524 | 80% | 0,1% | 39 | 44% | 578 (408–748) | 459 | 113% | 0.660 | 114% |
| - other | 55 | 75% | 114 (82–146) | 67 | 59% | 0,2% |  |  |  |  |  |  |  |

Notes: ^1^ %LSC: Percentage of products with labelled sodium content; ^2^ SCA: *Average sodium content of available prepacked foods* (95% confidence interval), ^3^ SCS: *Average sodium content of sold prepacked foods*, ^4^ SAR: Ratio between SCA and SCS; ^5^ Data from *Korošec et al.* 2014, with foods categorised according to *Dunford et al.* 2012; ^6^ p-value for comparison of *Average sodium content of available prepacked foods* [SCA] between both years using t-test/Mann-Whitney test; * p-value < 0.05.
